# Outpatient glucocorticoid use and COVID-19 outcomes: a population-based study

**DOI:** 10.1007/s10787-024-01474-3

**Published:** 2024-05-02

**Authors:** Almudena Rodríguez-Fernández, Irene Visos-Varela, Maruxa Zapata-Cachafeiro, Samuel Pintos-Rodríguez, Rosa M. García-Álvarez, Teresa M. Herdeiro, María Piñeiro-Lamas, Adolfo Figueiras, Ángel Salgado-Barreira, COVIDRUG Group, Irene Visos-Varela, Maruxa Zapata-Cachafeiro, Samuel Pintos-Rodríguez, Adolfo Figueiras, Rosendo Bugarín-González, Eduardo Carracedo-Martínez, Rosa M García-Álvarez, Francisco J González-Barcala, Teresa M Herdeiro, Martina Lema-Oreiro, Narmeen Mallah, Maria Piñeiro-Lamas, Manuel Portela-Romero, Angela Prieto-Campo, Almudena Rodriguez-Fernández, Marc Saez, Angel Salgado-Barreira, Margarita Taracido-Trunk

**Affiliations:** 1 Department of Preventive Medicine and Public Health, University of Santiago de Compostela, Rúa de San Francisco, S/N, 15782 Santiago de Compostela (A Coruña), Spain; 2 Consortium for Biomedical Research in Epidemiology and Public Health (CIBER en Epidemiología y Salud Pública - CIBERESP), Santiago de Compostela, Spain; 3 Health Research Institute of Santiago de Compostela (IDIS), University of Santiago de Compostela, Santiago de Compostela, Spain; 4 Service of Preventive Medicine and Public Health. Clinic Hospital of Santiago de Compostela, Santiago de Compostela, Spain; 5 Institute of Biomedicine (iBiMED), Department of Medical Sciences, University of Aveiro, 3810-193 Aveiro, Portugal

**Keywords:** Glucocorticoids, COVID-19, Hospitalization, Mortality, Real-world data

## Abstract

**Introduction:**

Owing to controversy information surrounds effect of glucocorticoids on the evolution of COVID-19, we evaluate the effects of outpatient glucocorticoid use on the severity and progression of COVID-19 and risk of infection and analyse the effect of window of exposure and dose.

**Methods:**

We conducted a population-based case − control study, involving 4 substudies: (i) Hospitalisation; (ii) Mortality, using subjects hospitalised with a PCR + as cases and subjects without a PCR + as controls; (iii) Progression, including subjects with a PCR + (hospitalised versus non-hospitalised); and (iv) Susceptibility, with all subjects with a PCR + and subjects without a PCR + . Adjusted odds ratios (ORa) and their 95% confidence intervals (95% CI) were calculated.

**Results:**

The outpatient glucocorticoid use was associated with an increased risk of hospitalisation (aOR 1.79; 95% CI 1.56–2.05), mortality (aOR 2.30; 95% CI 1.68–3.15), progression (aOR 1.69; 95% CI 1.43–2.00) and susceptibility (aOR 1.29, 95% CI 1.19–1.41). Furthermore, the effects was observed to be greater at higher doses and the closer that drug use approached the outcome date, with an almost fourfold increase in mortality among users in the previous month (aOR 3.85; 95% CI 2.63–5.62).

**Conclusions:**

According to the results of this real-world data study, outpatient glucocorticoid use should be considered in making decisions about intrahospital treatment.

**Supplementary Information:**

The online version contains supplementary material available at 10.1007/s10787-024-01474-3.

## Introduction

Glucocorticoids are a group of medications indicated for relieving inflammatory symptoms in many diseases, due to the pleiotropic effects of the glucocorticoid receptor on the immune system (Rhen and Cidlowski 2005). This same effect is responsible for some of these drugs’ side-effects, such as hyperglycaemia, hypertension, osteoporosis or increased risk of infection (Vandewalle et al. 2018). Although most of these adverse effects are linked to chronic use of glucocorticoids at high doses (Fardet and Fève 2014), they have also been observed in short-term users (Waljee et al. 2017) and at low doses (Galati et al. 2021).

Because the outbreak of the pandemic, glucocorticoids have played an important role in the management of COVID-19. Yet *controversy continues to surround their effect on clinical disease course*: on the one hand, their powerful *anti-inflammatory effect* ranks them as one of therapeutic options in the event of severe COVID-19 (Wagner et al. 2022), in that they reduce the release of cytokines (Villar et al. 2020); and on the other hand, their *immunosuppressive effect* could account for the fact that users of high doses of glucocorticoids have a higher risk of developing severe COVID-19 (Brenner et al. 2020).

Available evidence on the role of glucocorticoids in COVID-19 is based on small-sized studies (Liaquat et al. 2021) with very defined populations, such as subjects with autoimmune (Singh et al. 2021) or respiratory disease (Raj et al. 2022). Moreover, it is common for such studies to limit themselves to analysing a specific active ingredient (Velayos et al. 2021) or include only subjects who take doses above a given value (Ward et al. 2022; Ku et al. 2023). Furthermore, very few studies have analysed the risk of susceptibility, hospitalisation and mortality in the general population (Calderón-Parra et al. 2022; Ku et al. 2023) and none has studied all the outcomes in the same population-based cohort. Although some studies have analysed the effect of the dose taken (Strangfeld et al. 2021; Boteanu et al. 2022), we only located one in which the effect of different active ingredients were evaluated (Malekpour et al. 2023). Indeed, for some of the outcomes analysed, such as susceptibility, the studies are inconsistent, and as result, ambulatory glucocorticoid use has been associated, not only with a lower risk of infection (Liao et al. 2021), but also with a higher risk of infection (Singh et al. 2021).

To fill this knowledge gap, we conducted a study with the *main aim* of evaluating the effect of outpatient use of systemic corticoids on: (I) Severity of COVID-19 (in terms of risk of hospitalisation and risk of hospital mortality); (II) Progression to severe COVID-19; and (III) Susceptibility to SARS-COV-2 contagion. By way of *secondary aims*, we sought to analyse the variability of these risks according to window of exposure and defined daily dose (DDD) of glucocorticoids, and ascertain whether there were differences between the different active ingredients.

## Methods

### Study setting and population

The study was conducted in Galicia, a region situated in the north of Spain with 2.7 million inhabitants. Healthcare is provided by the Galician Health Service (GHS) which covers 98% of the regional population. The study population included all subjects over the age 18 years residing in Galicia, with GHS access. The study period was March to December 2020.

### Study design

We conducted a population-based, multiple case − control study (Rothman et al. 2008). Cases and controls were obtained from the same population, something that enabled us to establish a valid estimate of the prevalence of exposure and covariates in the source population. In the group of cases, exhaustive sampling was used to include all subjects with a PCR + test (hospitalised and non-hospitalised).

### Cases and controls

We conducted four case − control substudies, using different definitions of “case” and “control” (Table S1, see Supplementary data), to evaluate the effect of ambulatory glucocorticoid use on risk of severity (hospitalisation and mortality), progression to severe COVID-19, and susceptibility to SARS-CoV-2.

#### Case − control 1: severe COVID-19 outcomes-hospitalisation

To assess the risk of hospitalisation due to COVID-19, a case was defined as any patient with a positive PCR test (PCR +) admitted to a public hospital in Galicia due to COVID-19 (Table S1). Subjects hospitalised due to causes other than COVID-19 were eliminated for study purposes; to this end, we established a maximum difference of 10 days between the date of confirmation of diagnosis of COVID-19 and that of hospitalisation. For controls, we selected a random sample of subjects from the study population without a PCR + test, matched by age, sex, primary care service of reference, and status of health professional (Table S1). Up to 20 controls were defined for each case.

#### Case − control 2: severe COVID-19 outcomes–mortality

The effect on the risk of mortality was analysed by defining cases as all subjects who had died due to COVID-19 during hospitalisation at a GHS hospital. The control group was made up of case − control substudy 1 subjects (hospitalised), and was matched with the cases of this substudy (Table S1).

#### Case − control 3: progression to severe COVID-19 outcomes

To determine the effect of ambulatory glucocorticoid use on progression to severe disease, a case was defined as any patient hospitalised due to COVID-19, and the control group was made up of all patients with a PCR + test who did not require hospitalisation during the study period (Table S1).

In this substudy, the cases were not matched, something that was not associated with any increased risk of bias and did not affect the validity of the study, but only led to lower expected study effectiveness (Rothman et al. 2008; Rose and Van der Laan 2009).

#### Case − control 4: susceptibility to the virus

We assessed the risk of infection due to SARS-CoV-2, by defining cases as all patients with a positive PCR test regardless of whether or not they had been hospitalised, and the control group as the same subjects as those used in case − control substudy 1 who did not have a positive diagnosis of COVID-19. As in the case − control substudy on disease progression, these controls were not matched (Table S1).

### Data-source and collection

Automated data-extraction was performed independently by an information technology company from the GHS Data Analysis System (*Sistemas de informacion y Análisis Complejos/SIAC*) (Visos-Varela et al. 2023). As study covariates, we collected demographic anthropometric variables and comorbidities (hypertension, diabetes, chronic obstructive pulmonary disease (COPD), obesity, ischaemic heart disease, cerebrovascular accident, heart failure, atrial fibrillation, chronic renal failure, cancer, asthma, current smoker status, and number of treatments for chronic diseases) (Huber et al. 2013), and exposure to all medications prescribed and dispensed to each of the subjects in the 6 months preceding the index date.

#### Exposure

We defined the *main* variable of exposure as systemic glucocorticoid use (codes H02AB) in the *3 months* preceding the index date, which was set: as 10 days before confirmation of diagnosis of COVID-19 by a PCR + test; and for controls, as the same date as that of the cases with which they were matched. In addition, the following variables were used to achieve the secondary objectives:

1. to evaluate the impact of the *aetiological window*, we assessed use at 1, 2, 3 and 6 months preceding the index date;
2. to evaluate the effect of *dose*, we used the median as the cut point, with low use being classified as subjects who took doses below or equal to the median, and high use as subjects who took doses above the median;
3. to analyse the effect of the *different active ingredients* of glucocorticoids, we evaluated the use of dexamethsone (H02AB02), methlylprednisolone (H02AB04), prednisone (H02AB07), hydrocortisone (H02AB09), deflazacort (H02AB13) separately, and created an “Others” category which included bethamethasone (H02AB01), prednisolone (H02AB06) and triamcinolone (H02AB08)

### Statistical analysis

All the study outcomes were evaluated using generalised linear mixed models (Brown and Prescott 2006), due to the structure of the data and their advantages over conditional regression (Pinheiro and Bates 2000; Brown and Prescott 2006; Stroup 2012). The use of these models made it possible: (I) For matched and unmatched models to be analysed; (II) For the heterogeneity of initial clusters and time periods to be controlled for by the introduction of random terms; and (III) In strata in which there were no cases and controls, for the remaining subjects to count for analysis purposes.

In order to construct the model, the following aspects were considered: patient; case − control strata (for severity models); health centre; and pandemic wave. We used random-effects to assess the effect of the pandemic wave, and nested random-effects for patients, case and control strata, and health centre. To assess the effect of dose, active ingredient, and window of exposure, we performed complementary analyses. The results were expressed as adjusted odds ratios (aORs) with their 95% confidence intervals (CIs), with adjustments being made for the above-mentioned covariates.

Statistical significance was set at 0.05, and all statistical analyses were performed using the free R Statistical Software environment (version 4.1.2).

### Ethical aspects

The study was approved by the Galician Clinical Research Ethics Committee (*Comité de Ética de Investigación de Galicia*, reference 2020/349), certified by the Spanish Agency of Medicines and Medical Devices (*Agencia Española del Medicamento y Productos Sanitarios*), and conducted according to Helsinki Declaration principles and the prevailing legislation governing biomedical research. The study protocol is registered at the EU Electronic Register of Post-Authorisation Studies, EUPAS44587, and is available from https://www.encepp.eu/encepp/viewResource.htm?id=44588. The data were extracted and processed on an anonymised basis, thereby ensuring subjects’ confidentiality and privacy at all times.

## Results

**Fig. 1:**
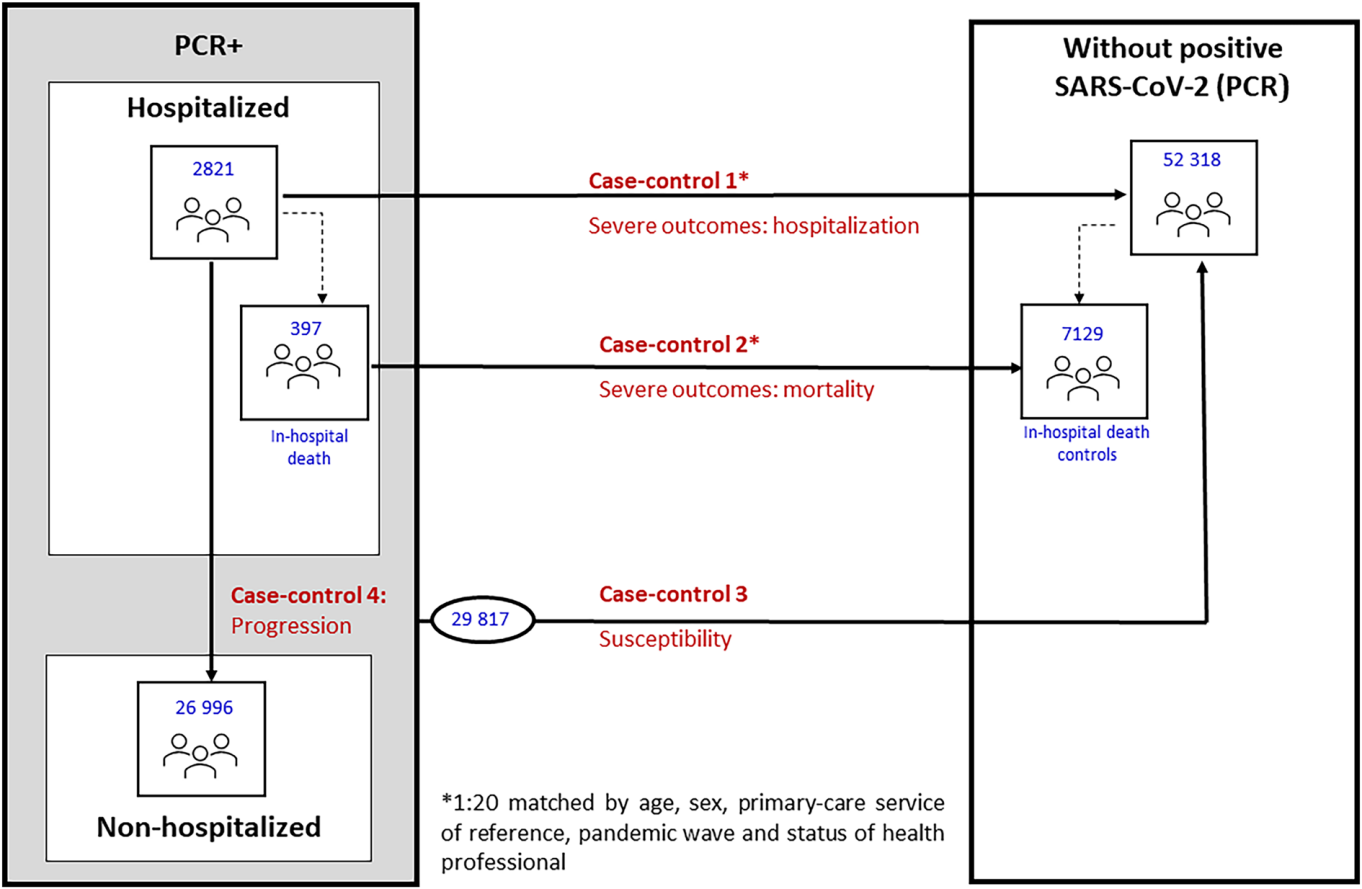
Population-based multiple case–control design

Our study covered a total of 82,315 subjects, namely, 2821 patients hospitalised due to COVID-19 (PCR +), 26,996 patients with a PCR + test and non-hospitalised, and 52,318 subjects without a PCR + test across the study period (Fig. 1). Table 1 shows the socio-demographic and clinical variables of each cohort. Hypertension and diabetes were the most frequent comorbidities in the group of hospitalised patients. Median use of glucocorticoids among users totalled 0.34 DDDs/day.

**Table 1:** Demographic and clinical characteristics of the groups

	PCR + Hospitalised^a^ (*n* = 2821) *n* (%)	PCR + Non-hospitalised (*n* = 26,996) *n* (%)	Non PCR + (*n* = 52,318) *n* (%)
Sex			
Male	1457 (51.6)	11,217 (41.6)	26,998 (51.6)
Female	1364 (48.4)	15,779 (58.4)	25,320 (48.4)
Age, median (IQR^b^)	74 (60.0 – 85.0)	47 (33.0–63.0)	73 (60.0–84.0)
Health professional	78 (2.8)	1238 (4.6)	1203 (2.3)
Comorbidities			
Hypertension	1639 (58.2)	6208 (23.0)	26,292 (50.3)
Diabetes	782 (27.8)	2519 (9.3)	10,233 (19.6)
COPD	369 (13.1)	759 (2.8)	4305 (8.2)
Obesity	830 (29.5)	3960 (14.7)	10,104 (19.3)
Ischaemic heart disease	326 (11.6)	865 (3.2)	4479 (8.6)
Cerebrovascular accident	277 (9.8)	867 (3.2)	3631 (6.9)
Heart failure	430 (15.3)	678 (2.5)	3780 (7.2)
Atrial fibrillation	425 (15.1)	1076 (4.0)	5405 (10.3)
Chronic renal failure	403 (14.3)	712 (2.6)	4059 (7.8)
Cancer	475 (16.9)	1755 (6.5)	7277 (13.9)
Asthma	267 (9.5)	2170 (8.0)	3070 (5.9)
Current smoker	737 (26.1)	4108 (15.2)	7842 (15.0)

^a^Hospitalised: further information on the case–control definition is presented in Table S1 of the supplementary material

^b^IQR = interquartile range; COPD = chronic obstructive pulmonary disease

### Severe COVID-19 outcomes

#### Risk of hospitalisation

Risk of hospitalisation was analysed on the basis of 2821 subjects hospitalised with a PCR + test, and a random sample of 52,318 subjects without a PCR + test (Table 1).

**Fig. 2:**
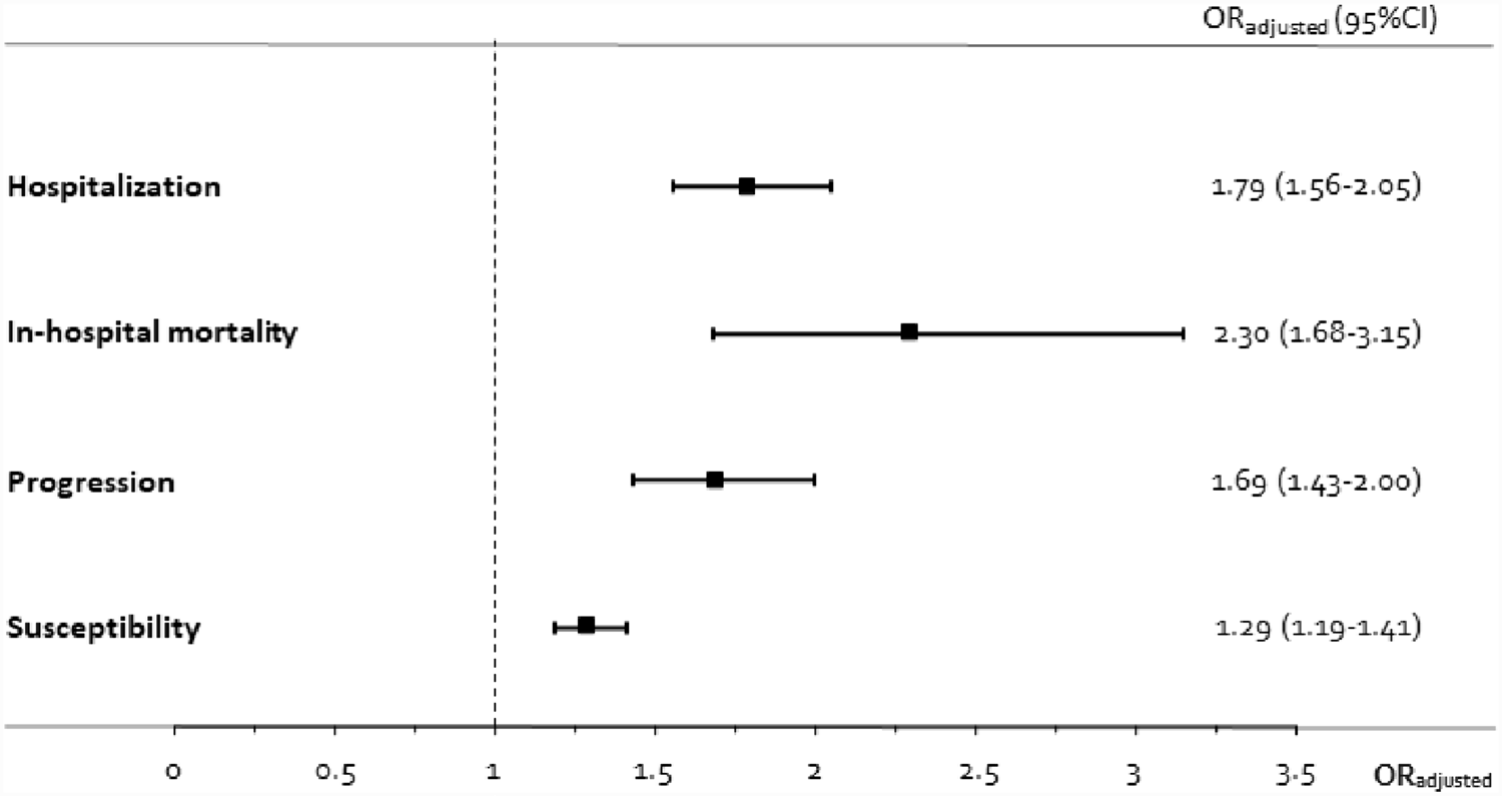
Adjusted odds-ratio of the association between outpatient glucocorticoids and COVID-19 severe outcomes

Glucocorticoid use in the *3 months preceding diagnosis* of COVID-19 (Fig. 2) increased the risk of hospitalisation due to COVID-19 (aOR 1.79; 95% CI 1.56–2.05). Moreover, this risk rose progressively (Table 2), the closer that drug use approached the index date (aOR 2.53; 95% CI 2.15–2.97).

**Table 2:** Risk of hospitalization, mortality, progression, and susceptibility, considering different windows of exposure to glucocorticoids

Model	Cases^a^ *n* (%)	Controls^a^ *n* (%)	Adjusted OR^b^	*P* value
**Hospitalization**	***n*=2821**	***n*=52318**		
6 months	393 (13.9)	3469 (6.6))	1.68 (1.49–1.90)	<0.001
3 months	299 (10.6)	2425 (4.6)	1.79 (1.56–2.05)	<0.001
2 months	262 (9.3)	1912 (3.7)	2.00 (1.74–2.31)	<0.001
1 months	206 (7.3)	1194 (2.3)	2.53 (2.15–2.97)	<0.001
**Mortality**	***n*=397**	***n*=7129**		
6 months	78 (19.6)	562 (7.9)	1.95 (1.46–2.61)	<0.001
3 months	64 (16.1)	386 (5.4)	2.30 (1.68–3.15)	<0.001
2 months	58 (14.6)	295 (4.1)	2.74 (1.98–3.81)	<0.001
1 months	44 (11.1)	162 (2.3)	3.85 (2.63–5.62)	<0.001
**Progression**	***n*=2821**	***n*=26996**		
6 months	393 (13.9)	1246 (4.6)	1.57 (1.36–1.82)	<0.001
3 months	299 (10.6)	822 (3.0)	1.69 (1.43–2.00)	<0.001
2 months	262 (9.3)	635 (2.4)	1.91 (1.59–2.28)	<0.001
1 months	206 (7.3)	421 (1.6)	2.14 (1.74–2.64)	<0.001
**Susceptibility**	***n*=29817**	***n*=52318**		
6 months	1639 (5.5)	3469 (6.6)	1.26 (1.17–1.35)	<0.001
3 months	1121 (3.8)	2425 (4.6)	1.29 (1.19–1.41)	<0.001
2 months	897 (3.0)	1912 (3.7)	1.32 (1.2–1.44)	<0.001
1 months	627 (2.1)	1194 (2.3)	1.49 (1.33–1.66)	<0.001

^a^Cases and Controls: further information on the case–control definition of each substudy is presented in Table S1 of the supplementary material

^b^OR = odds ratio- Adjusted for: sex, age, and comorbidities (hypertension, diabetes, chronic obstructive pulmonary disease, obesity, ischaemic heart disease, cerebrovascular accident, heart failure, atrial fibrillation, chronic renal failure, cancer, asthma, current smoker), current use of other pharmacological treatment and number of treatments for chronic diseases

Tables 3, 4 show the results of the analysis by dose and active ingredient. An increased risk of hospitalisation was observed for all doses (≤ 0.34 DDDs/day: aOR 1.70 [95% CI 1.41–2.05] and > 0.34 DDDs/day: aOR 1.87 [95% CI 1.57–2.23]) and was also observed in users for each of the active ingredients analysed separately.

**Table 3:** Risk of hospitalization, mortality, progression, and susceptibility considering DDD/day glucocorticoids consumption 3 months before

Model	Cases^a^ *n* (%)	Controls^a^ *n* (%)	Adjusted OR^b^	*P *value
**Hospitalization**	***n*=2821**	***n*=52318**		
Glucocorticoids–non consumption	2522 (89.4)	49893 (95.4)	–	–
Glucocorticoids–low consumption (≤0.34 DDDs/day)^c^	134 (4.8)	1202 (2.3)	1.70 (1.41–2.05)	<0.001
Glucocorticoids–high consumption (>0.34 DDDs/day)	165 (5.8)	1223 (2.3)	1.87 (1.57–2.23)	<0.001
**Mortality**	***n*=397**	***n*=7129**		
Glucocorticoids–non consumption	333 (83.9)	6743 (94.6)	–	–
Glucocorticoids–low consumption (≤0.34 DDDs/day)	26 (6.5)	196 (2.7)	1.81 (1.15–2.85)	0.010
Glucocorticoids–high consumption (>0.34 DDDs/day)	38 (9.6)	190 (2.7)	2.80 (1.89–4.15)	<0.001
**Progression**	***n*=2821**	***n*=26996**		
Glucocorticoids–non consumption	2522 (89.4)	26174 (97.0)	–	–
Glucocorticoids–low consumption (≤0.34 DDDs/day)	134 (4.8)	427 (1.6)	1.63 (1.29–2.06)	<0.001
Glucocorticoids–high consumption (>0.34 DDDs/day)	165 (5.8)	395 (1.5)	1.75 (1.40–2.19)	<0.001
**Susceptibility**	***n*=29817**	***n*=52318**		
Glucocorticoids–non consumption	28696 (96.2)	49893 (95.4)	–	–
Glucocorticoids–low consumption (≤0.34 DDDs/day)	561 (1.9)	1202 (2.3)	1.23 (1.09–1.38)	<0.001
Glucocorticoids–high consumption (>0.34 DDDs/day)	560 (1.9)	1223 (2.3)	1.36 (1.21–1.53)	<0.001

^a^Cases and Controls: further information on the case − control definition of each substudy is presented in Table S1 of the supplementary material

^b^Adjusted OR = odds ratio- Adjusted for: sex, age, and comorbidities: hypertension, diabetes, chronic obstructive pulmonary disease, obesity, ischaemic heart disease, cerebrovascular accident, heart failure, atrial fibrillation, chronic renal failure, cancer, asthma, current smoker, current use of other pharmacological treatment and number of treatments for chronic diseases

^c^DDD: defined daily dose, cut of point: median

**Table 4:** Risk of hospitalization, mortality, progression, and susceptibility, considering different glucocorticoids (ATC code) 3 month before

Model	Cases^a^ *n* (%)	Controls^a^ *n* (%)	Adjusted OR^b^	*P* value
**Hospitalization**	***n*=2821**	***n*=52318**		
Dexamethasone (H02AB02)	22 (0.8)	111 (0.2)	2.72 (1.69–4.36)	<0.001
Methylprednisolone (H02AB04)	32 (1.1)	197 (0.4)	2.40 (1.63–3.53)	<0.001
Prednisone (H02AB07)	193 (6.8)	1502 (2.9)	1.74 (1.48–2.05)	<0.001
Hydrocortisone (H02AB09)	8 (0.3)	50 (0.1)	2.16 (1.00–4.66)	0.049
Deflazacort (H02AB13)	58 (2.1)	561 (1.1)	1.4 (1.06–1.86)	0.019
Other glucocorticoids^c^ (H02AB01/H02AB06/H02AB08)	2 (0.1)	74 (0.1)	0.43 (0.11–1.78)	0.245
**Mortality**	***n*=397**	***n*=7129**		
Dexamethasone (H02AB02)	3 (0.8)	15 (0.2)	2.46 (0.68–8.88)	0.170
Methylprednisolone (H02AB04)	9 (2.3)	34 (0.5)	3.17 (1.40––7.17)	0.006
Prednisone (H02AB07)	47 (11.8)	230 (3.2)	2.78 (1.94–3.99)	<0.001
Hydrocortisone (H02AB09)	2 (0.5)	5 (0.1)	5.46 (0.95–31.47)	0.058
Deflazacort (H02AB13)	10 (2.5)	100 (1.4)	1.19 (0.59–2.38)	0.625
Other glucocorticoids^c^ (H02AB01/H02AB06/H02AB08)	0 (0)	11 (0.2)	-	-
**Progression**	***n*=2821**	***n*=26996**		
Dexamethasone (H02AB02)	22 (0.8)	42 (0.2)	2.13 (1.14–3.96)	0.017
Methylprednisolone (H02AB04)	32 (1.1)	66 (0.2)	1.96 (1.20–3.20)	0.008
Prednisone (H02AB07)	193 (6.8)	469 (1.7)	1.77 (1.44–2.19)	<0.001
Hydrocortisone (H02AB09)	8 (0.3)	20 (0.1)	1.83 (0.72–4.68)	0.206
Deflazacort (H02AB13)	58 (2.1)	224 (0.8)	1.17 (0.82–1.65)	0.386
Other glucocorticoids^c^ (H02AB01/H02AB06/H02AB08)	2 (0.1)	29 (0.1)	0.54 (0.12–2.37)	0.415
**Susceptibility**	***n*=29817**	***n*=52318**		
Dexamethasone (H02AB02)	64 (0.2)	111 (0.2)	1.29 (0.91–1.83)	0.152
Methylprednisolone (H02AB04)	98 (0.3)	197 (0.4)	1.47 (1.12–1.94)	0.006
Prednisone (H02AB07)	662 (2.2)	1502 (2.9)	1.26 (1.14–1.41)	<0.001
Hydrocortisone (H02AB09)	28 (0.1)	50 (0.1)	1.27 (0.76–2.13)	0.368
Deflazacort (H02AB13)	282 (0.9)	561 (1.1)	1.36 (1.15–1.61)	<0.001
Other glucocorticoids^c^ (H02AB01/H02AB06/H02AB08)	31 (0.1)	74 (0.1)	0.83 (0.52–1.33)	0.450

^a^Cases and Controls: further information on the case–control definition of each substudy is presented in Table S1 of the supplementary material

^b^Adjusted OR = odds ratio- Adjusted for: sex, age, and comorbidities (hypertension, diabetes, chronic obstructive pulmonary disease, obesity, ischaemic heart disease, cerebrovascular accident, heart failure, atrial fibrillation, chronic renal failure, cancer, asthma), current smoker, current use of other pharmacological treatment and number of treatments for chronic diseases

^c^Other glucocorticoids: betamethasone, prednisolone, and triamcinolone

#### Risk of mortality

The risk of mortality was determined on the basis of 397 PCR + patients who died in hospital and 7129 controls matched without a PCR + test. The risk of mortality was significantly higher among glucocorticoid users (aOR 2.30; 95% CI 1.68–3.15) (Fig. 2), with the highest risk being observed among those who had used glucocorticoids in the preceding month (aOR 3.85; 95% CI 2.63–5.62) (Table 2).

Patients who took a higher dose of glucocorticoids had a higher risk of dying (aOR 2.80; 95% CI 1.89–4.15) than did non-users (Table 3). Of the active ingredients studied (Table 4), hydrocortisone was the active ingredient for which the highest mortality risk was observed, but the results were not significant (aOR 5.46; 95% CI 0.95–31.47). Methylprednisolone and prednisone increased the mortality risk significantly (aOR 3.17 [95% CI 1.40–7.17] and aOR 2.78 [95% CI 1.94–3.99] respectively).

### Progression to severe COVID-19 outcomes

The risk of progression of SARS-Cov2 infection to severe stages was analysed using data on 2821 hospitalised patients with a PCR + test and 26,996 controls (non-hospitalised with a PCR + test). Overall, glucocorticoid treatment significantly increased the risk of progression to severe COVID-19 among subjects with a positive diagnosis (aOR 1.69; 95% CI 1.43–2.00) (Fig. 2).

Furthermore, this risk rose progressively as drug use approached closer to the date of hospitalisation, rising to 2.14 (95% CI 1.74–2.64) among subjects with use in the preceding month (Table 2). This significant increase in risk was observed for glucocorticoid users of low (aOR 1.63; 95% CI 1.29–2.06]) and high doses alike (aOR 1.75; 95% CI 1.40–2.19) (Table 3). Dexamethasone was the active ingredient with the highest risk of progression to severe COVID-19 (aOR 2.13; 95% CI 1.14–3.96) (Table 4).

### Susceptibility to the virus

The risk of infection due to SARS-CoV-2 was assessed on the basis of 29,817 subjects with a PCR + test (2821 hospitalised and 26,996 non-hospitalised), and 52,318 subjects without a PCR + test (Table 1). In the analysis by window of exposure (Table 2), glucocorticoids were observed to increase the risk of susceptibility, the closer their use approached the index date, ranging from the previous 6 months (aOR 1.26; 95% CI 1.17–1.35) to the preceding month (aOR 1.49; 95% CI 1.33–1.66). In the analysis by active ingredient (Table 4), the highest risk was associated with use of methylprednisolone (aOR 1.47; 95% CI 1.12–1.94).

## Discussion

This large-sized Real-World Data (RWD) study suggests that outpatient glucocorticoid treatment is consistently associated with a higher risk of severe COVID-19 outcomes, and that this effect is seemingly independent of the active ingredient. Furthermore, our results suggest that, as use approaches the index date and as the dose increases, the risk of susceptibility, progression, hospitalisation, and mortality due to COVID-19 rises. Specifically, risk of death is almost fourfold higher among subjects who use glucocorticoids in the previous month.

The increased risk found in our study for *different outcomes* is in line with the findings of some studies conducted on patients with autoimmune or respiratory disease, among whom ambulatory glucocorticoid use has been associated with an increased risk of susceptibility (Singh et al. 2021), hospitalisation (Brodin et al. 2022), disease progression (Ungaro et al. 2021) and mortality (Calderón-Parra et al. 2022; Malekpour et al. 2023). In addition, our results suggest that this effect is independent of the approved active ingredient, and is observed for both high and low doses (≤ 0.34 DDDs/day), unlike other studies on patients with autoimmune disease, in which the greatest risk of susceptibility and severe COVID-19 outcomes was exclusively reported for users of high doses of glucocorticoids (Gianfrancesco et al. 2020; Shin et al. 2021).

The increased *susceptibility* observed in our study (aOR 1.29; 95% CI 1.19–1.41, *p* < 0.001*) is consistent with* the increased risk of infections found by Dixon et al. in their meta-analysis (Dixon et al. 2011). This could be accounted for by the effect of these medications on the suppression of cellular immunity and alterations in phagocyte function (Cutolo et al. 2008). Insofar as *disease progression* is concerned, other studies have reported that SARS-CoV-2 infection could present with more severe symptoms in patients with autoimmune (Khan et al. 2021) or respiratory disease (Adir et al. 2021) treated with glucocorticoids, due in part to the reduction in SARS-CoV-2 RNA clearance (FakhriRavari et al. 2021). Our results would support this hypothesis, since risk of *disease progression* (aOR 1.69; 95% CI 1.43–2.00, *p* < 0.001) is greater among patients with ambulatory glucocorticoid use than among non-users.

The 1.8-fold higher risk of *hospitalisation* (aOR 1.79; 95% CI 1.56–2.05, *p* < 0.001) observed in our study could be due to suppression of immune T-cell response, which would hinder initial diagnosis of COVID-19 (Migita et al. 2013), along with the harmful effect of treatment with glucocorticoids in the initial stages (Wagner et al. 2022). In this respect, this increased risk of *hospitalisation* shows a high degree of internal consistency in our study, since we also found an increased risk of *susceptibility* (aOR 1.29; 95% CI 1.19–1.41, *p* < 0.001*) and disease progression* (aOR 1.69 95% CI 1.43–2.00, *p* < 0.001).

Our results show that the risk of *mortality* due to COVID-19 among hospitalised patients with ambulatory glucocorticoid treatment in the preceding month is fourfold higher than that of non-users (aOR 3.85; 95% CI 2.63–5.62, *p* < 0.001). This higher risk of mortality is in line with what has been suggested in the previous studies but, unlike ours, this evidence is based on the studies conducted on very defined populations, such as subjects with autoimmune (Ward et al. 2022) or respiratory disease (Adir et al. 2021), users of high doses (Brodin et al. 2022), hospitalised patients (Suárez-García et al. 2021), and subjects with a PCR + test (Ku et al. 2023). This important risk of *mortality* could be accounted for: (i) By the increased risk of developing serious (George et al. 2020) or invasive fungal infections (Lionakis and Kontoyiannis 2003); (ii) By the harmful effect of glucocorticoid treatment in the initial stages of the disease (Li et al. 2020); and (iii) By the lack of effectiveness of glucocorticoid treatment in hospitalised patients, due to possible resistance to glucocorticoids (Bruscoli et al. 2022), as a possible consequence of prolonged exposure to inflammatory cytokines (Quax et al. 2013).

In our study, the risk of *mortality* among users in the previous 3 months (aOR 2.30; 95% CI 1.68–3.15, *p* < 0.001) would appear to be higher than the risk of hospitalisation (aOR 1.79; 95% CI 1.56–2.05, *p* < 0.001). This could be accounted for by possible resistance to glucocorticoid treatment (Barnes and Adcock 2009) among inpatients or the appearance of side-effects associated with prolonged exposures to or high doses of glucocorticoids (Rhen and Cidlowski 2005). This lack of a glucocorticoid-induced effect on hospitalised patients taking glucocorticoids could give rise to a higher risk of mortality. It has been suggested that prolonged exposures to inflammatory cytokines, such as that which occurs in cases of severe COVID-19, could develop resistance to glucocorticoids (Quax et al. 2013).

The effects observed on all the outcomes analysed in our study are greater when they are associated with *higher doses and use closer to the time of infection*. This finding lends great internal consistency to our results, and suggests that the effect of glucocorticoids is maintained throughout the clinical disease course and favours progression to severe clinical profiles. It has been suggested that this could be due to the fact that use of glucocorticoids might delay the manifestation of the initial symptoms of COVID-19, (Calderón-Parra et al. 2022) or alternatively, to the harmful effect that these drugs have on the initial stages of the disease (Li et al. 2020). Moreover, as our results show, the closer glucocorticoid use is to the index date, and hence to infection, the higher the risk for all outcomes. This harmful effect in the initial disease stages, has also been suggested in other viral infections, such as that caused by SARS-CoV (Lee et al. 2004) or influenza-virus associated pneumonia (Ni et al. 2019).

### Strengths and limitations of the study

Our study’s main strength lies in the *internal consistency of our data* for all outcomes studied. Another aspect of note is its *large sample size*, which made it possible to carry out separate analyses of the effect of dose, window of exposure and different glucocorticoids, and thereby rule out differences between them for the first time. Similarly, the fact that this is an RWD study and includes *all* the diagnosed cases of COVID-19 means that there is *no selection bias*, and that we were able to adjust the outcomes for socio-demographic *variables*, comorbidities, and previous treatments with other drugs, which might have otherwise confounded the effect observed. Lastly, the variables of exposure were obtained from dispensing rather than from prescription data, which reduces the risk of misclassification.

Our study has a series of limitations which should be considered when it comes to interpreting the results. Firstly, the fact that it is an *observational study* with secondary databases means that one cannot rule out the existence of confounding factors not included in our analysis or the possibility of misclassification in some variables due to prescriptions issued by private healthcare sources. Secondly, it could be thought that not having information about some indications for glucocorticoids could lead to confounding by *indication bias*, since glucocorticoids are used as treatment for chronic inflammatory and immune diseases (Barnes 2011), such as COPD, associated with a higher risk of severe outcomes (Christenson et al. 2022). We do not consider this to be a limitation, however, since an important part of indications for glucocorticoids are either not related to an increased risk (Choi and Seeger 2005), are not associated with a specific disease (Fardet et al. 2011), or are included in the variables of adjustment of our results (Waljee et al. 2017). Thirdly, the *lack of matching* in the susceptibility and disease progression substudies might be a limitation. That said, this lack of matching in the case − control substudies implies a decrease solely in efficacy, and not in the overall validity of the results (Rothman et al. 2008; Rose and van der Laan 2009). Lastly, it is likely that in the group of subjects without a PCR + test, asymptomatic subjects or subjects with mild symptomatology may have been included due to the *difficulty of access to COVID-19 diagnostic tests* in Spain, especially at the outbreak of the pandemic.

The increased risk of severe COVID-19 outcomes should be taken into account by clinicians when it comes to prescribing these medications, especially at high doses. Due to the many side-effects associated with glucocorticoids (Rhen and Cidlowski 2005; Moghadam-Kia and Werth 2010; Waljee et al. 2017), their use should be optimised by employing the smallest possible doses. Our results will add evidence to this recommendation, since the risk of severe outcomes and susceptibility increases among users of high doses.

Our results suggest that ambulatory use of systemic glucocorticoids is associated with an increased risk of severe COVID-19 outcomes, regardless of the active ingredient consumed, even at low doses. The effect of ambulatory glucocorticoid use increases as the clinical course of the disease advances. Furthermore, all risks become consistently higher as drug use approaches closer to the index date. Given the large effect magnitude found in our study and the fact that glucocorticoids are one of the medications used in intrahospital treatment of COVID-19, our results suggest that it would be of interest to conduct a specific intrahospital follow-up of patients who have consumed glucocorticoids on an ambulatory basis.

## Supplementary Information

Below is the link to the electronic supplementary material.

Supplementary file1 (DOCX 15 KB)

## Data Availability

The data sets generated and analysed during the current study are not publicly available due to Galician Public Health System restrictions.
