# Supplementary material for: Outpatient glucocorticoid use and COVID-19 outcomes: a population-based study
Source: Inflammopharmacology. 2024 May 2;32(4):2305–15. doi: 10.1007/s10787-024-01474-3 (PMC11300658; doi:10.1007/s10787-024-01474-3)
Supplement: Supplementary file 1 — Supplementary file1 (DOCX 15 KB) [file 10787_2024_1474_MOESM1_ESM.docx]

**Table S1**. Summary of the 4 case-control substudies.

| **Case-control** | **Aim** | **Cases** | **Controls** | **Matching** | **Index date** |
| --- | --- | --- | --- | --- | --- |
| - 1. **Severe COVID-19 outcomes - hospitalization** | To assess the effect of glucocorticoids on risk of hospitalization for COVID-19. | All subjects >18 years admitted for COVID-19 (PCR+) in a GHS hospital (N=2821). | Subjects who did not present PCR+, matched with cases (N=52 318). | Yes* | Cases: 10 days prior to the PCR+  Controls: the same as for its matched case. |
| - 1. **Severe COVID-19 outcomes - mortality** | To assess the effect of glucocorticoids on risk of mortality in patients with COVID-19. | All subjects >18 years hospitalized for COVID-19 who died during admission to any of the GHS hospitals in 2020 (N=397). | Subjects who did not present PCR+, matched with cases (N=7129). | Yes* | Cases: 10 days prior to the PCR+  Controls: the same as for its matched case. |
| 1. **Progression to severe COVID-19 outcomes** | To evaluate the effect of glucocorticoids on disease progression to more severe stages that might require hospital admission. | Same cases as those in the Case-control 1 substudy (N=2821). | All patients with diagnosis of COVID-19 confirmed by PCR, who did not require hospitalization  (N=26 996). | No | Cases: 10 days prior to the PCR+  Controls: 10 days prior to the PCR+. |
| 1. **Susceptibility to the virus** | To establish the impact of glucocorticoids on risk of infection. | All subjects over the age of 18 years with diagnosis of COVID-19, confirmed by PCR (both hospitalized and non-hospitalized)  (N=29 817). | Same controls as those in the Case-control 1 substudy  (N=52 318). | No | Cases: 10 days prior to the PCR+  Controls: the same as for cases of Case-control 1 substudy. |

GHS = Galician Health Service. *1:20 matched by age, sex, primary-care service of reference, pandemic wave and status of health professional.
